# COVID-19, new challenges to human safety: a global review

**DOI:** 10.3389/fpubh.2024.1371238

**Published:** 2024-03-14

**Authors:** Saierdaer Aikebaier

**Affiliations:** Department of Public Administration, School of Public Affairs, Xiamen University, Xiamen, Fujian, China

**Keywords:** sustainable human safety, safety crack, DV, IPV, crime, COVID-19

## Abstract

In the context of sustainable human development, human safety has gradually shifted from traditional state and political conflict to social conflict and horizontal inequality, and the pandemic has exacerbated this variation risk. This narrative review includes literature from 40 countries on five continents since 2020, explored and tidy up the impacts of pandemics on human safety based on three perspectives: personal safety, family safety and social safety, refined the macroscopic concept of human safety. The comprehensibility of the global review conclusions is enhanced by combining it with Maslow’s hierarchy of needs. Finally, some novel and comparative results are included to broaden the understanding of the impact of the pandemic, and help policymaker better understand human safety changes from a new perspective.

## 1 Introduction

The 1994 Human Development Report state (1): the human safety approach refocused the safety debate from territorial safety to the safety of people. Human safety is about living free from want, free from fear and free from humiliation. It is about protecting what we humans value most in our lives. The United Nations General Assembly (UNGA) reflected a consensus that human safety is “the right of people to live in freedom and dignity, free from poverty and despair. All people, especially the most vulnerable, are entitled to freedom from fear and freedom from want, with equal opportunities to enjoy all their rights and fully develop their human potential.” Recently, the United Nations Development Program, in it’s Special Report (2022) has shown that humanity is facing increasingly serious multiple and overlapping threats, and exacerbated by pandemic (2).

According to the Special Report (2), the disconnect between human development and safety may be a by-product of pursuing a developmental approach, coupled with the legacy of historical injustices such as colonization, development has not benefited all, and even in some cases has left some groups behind. An approach to development that focuses most of its attention on economic growth, and much less on equitable human development, leads to serious and growing inequalities and puts increasing pressure on the planet. The pandemic exacerbated this incomplete impact. The pandemic has now affected everyone, threatening every dimension of our well-being and creating an acute sense of fear across the globe, combined with rising geopolitical tensions, widening inequalities, democratic backsliding and destructive weather events (linked to climate change), threatens to reverse decades of development gains and further derail progress toward the sustainable development goals. The pandemic has affected almost everyone and has become a full-blown crisis of human safety and development. The most tragic impact has been a global death toll of more than 10 million (excess mortality in 2020–2021). But the impact goes far beyond this harrowing record. Most countries have experienced acute recessions. And with the outbreak, a growing sense of insafety has taken hold. It is estimated that six out of seven people around the world feel insecure in the years leading up to a pandemic. Not only is this sense of insafety high, but it has also increased in most countries for which data are available, including spikes in some of the countries with the highest human development indexes.

The pandemic highlights the interconnectedness of factors affecting human safety and reveals new cumulative threats to human safety—violent conflict and rising horizontal inequalities (2). At present, the concept of violent conflict is gradually shifting from the traditional political confrontation between states to a people-centered conflict between individuals and societies, encompassing the stabilization of social order and the safety of individuals. For these two main points, this review synthesizes the impact of pandemic on personal health in terms of personal spirituality and life safety. An overview of the impact of pandemic on social order in terms of changes in social crime. In response to the horizontal inequality to human safety, this review focuses on changes in gender inequality during the pandemic. It is noteworthy that the ongoing pandemic seems to have triggered an increase in domestic and social violence (with women and children being the main victims). Conflict and violence may force people to leave their homes, exposing them to further threats.

**Figure 1:**
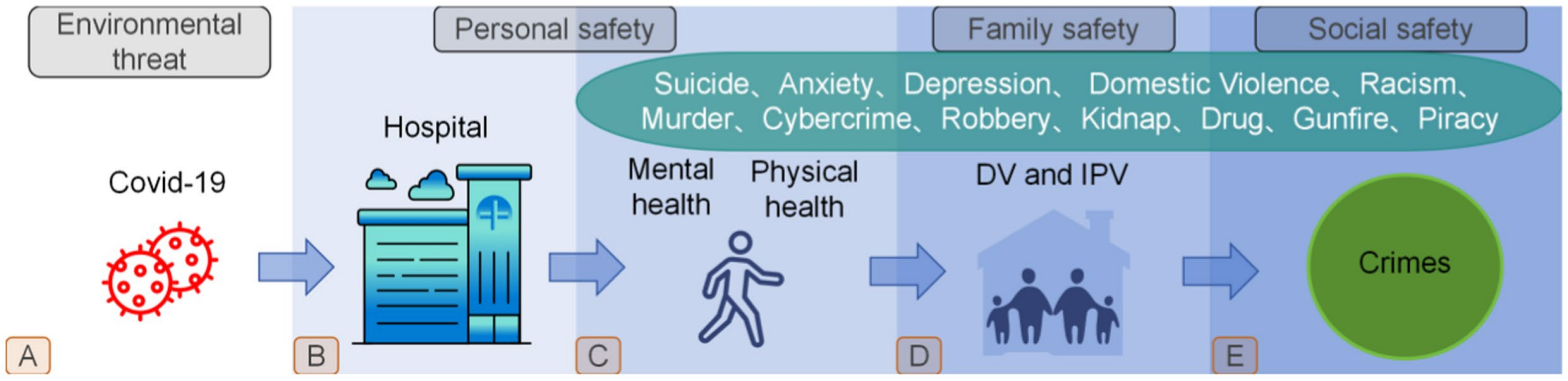
New human safety challenges due to COVID-19.

Based on the above background, this study concludes that human safety requires consideration of overlapping threats and systemic response that adapts to changing circumstances (Figure 1). According to the Maslow’s hierarchy of needs, only when the life existence been protected, then will move on to higher safety needs. Thus, in addition to studying the impact that the pandemic have had on human survival (processes A to B in Figure 1), it is important to understand that the human being is a complex synthesis, the pandemic also have far-reaching effects on human safety beyond the need for survival, researchers not only should focus on the most basic human needs, but also on spiritual and a range of other human safety needs that overlap in complexity. Unfortunately, it is clear from the literature cited in this review that while most studies have analyzed the impact of pandemics on suicides, domestic violence, robberies, shootings, and so on, these have been exploratory studies of a single target or a specific group of people; what is lacking is a review that combines these studies with human security research. Therefore, this narrative review aims to provide an overview of the impact of pandemics on human safety in the form of a synthesis and to summarize these non-linearly overlapping safety factors into three main dimensions: personal, family and social safety (Processes C, D and E in Figure 1) to help us better understand the far-reaching impact of the pandemic and to provide policy makers with a kind of early warning to formulate better adapted policies to address some under-appreciated but highly deserved societal problems.

## 2 Methods and data synthesis

Given the early stage in the evolving literature on this topic, this review performed a literature search in Pubmed and Web of Science electronic databases up to 31 May 2023 using “COVID-19” as the primary keyword, “violence,” “crime,” “depression,” “anxiety” and “suicide” as secondary keywords, and the reference lists of the selected literature were reviewed. Then 308 publications were identified by deleting the same. Based on this, the author performed a comprehensive reading to these literature, and screened them according to the following inclusion criteria: (1) the study need to include explicit positive or negative perspectives; (2) the literature need to include a variety of country and research target; (3) the focus of the studies should be on COVID-19-related impacts; (4) the research target need to be representative a group, community, or country. A study was excluded if it (1) used mainly qualitative methods and had neutral conclusions, (2) mainly modeling or methodological innovations without focusing on practical impacts. Ultimately, based on the above criteria, the author assessed all 308 publications, and evaluated the authority of these publishers empirically, a total of 94 full publications were selected to include in this narrative review.

## 3 Results

Based on the literature, it is known that the human safety challenges resulting from COVID-19 were found to be intricate, and it’s involved almost every continent (Table 1). In order to provide the reader with a better understanding of such intricate issues, this study categorizes such issues into three categories: personal security, family security and social security, a categorization that has the advantage of making the macro-concept of human security more nuanced and easier to understand. Personal safety focuses primarily on individuals, outlining changes in both psychiatric and life during the pandemic, identifies trends in the fluctuation of these changes over time, highlights which populations are most affected, and reveals gender differences. Family safety is formed on the basis of personal safety, mainly to show what happens when two different safety factors are combined, and whether personal safety is strengthened or challenged? Social safety, is an interconnected system of individual and family, although family safety constitutes the vast majority of social safety. However, the possibility that individuals may be separated from their families and pose a threat to social safety cannot be ignored. Therefore, the social safety are synthesized into a combination of individual and family safety, which points to a larger human safety structure.

**Table 1:** Human safety issues arising from the pandemic.

Continent	Nation	COVID-19 caused safety issue	References
America	Canada, United States, Trinidad and Tobago, Colombia, Peru, Mexico, Brazil	Depression and anxiety, Youth suicide, IPV, Societal crime, Gun violence, Robberies, Kidnappings, Homicides, Organized crime, Shootings, Racial discrimination	(3–17)
Europe	Germany, Hungary, Poland, Croatia, Austria, Sweden, Lithuania, Spain, France, Denmark, United Kingdom, Belgium, Turkey, Norway, England, Wales	Anxiety and depression, Suicide, Young female suicide, IPV, DV, Homicide, Drugs, Cybercrime, Hacking	(18–40)
Asia	South Korea, Bangladesh, Japan, Nepal, Russia, Palestine, Iraq, Jordan, Israel	Depression and anxiety, Suicidal ideation, Youth suicide, Domestic violence, Sexual assault, Violence against women, Violent crime, Property crime, Drug trafficking	(41–62)
Africa	Nigeria, Tunisia, Uganda, Kenya, Malawi, Zimbabwe	Mental health, Youth vulnerability, Women’s aggression, IPV, Night-time crime, Illicit drugs, piracy incidents, Racial discrimination	(63–71)
Oceania	Australia, New Zealand	Suicide, Domestic violence	(72–74)

### 3.1 Personal safety

The most direct damage to mankind from the pandemic is the biological attack on each individual (devastation the human immune system), which is a physical damage. However, man is an emotional being with an independent consciousness, and the need for mental safety cannot be ignored. In addition to the severe physical damage that the pandemic has inflicted on the human body, it has also had an even more severe long-term negative impact on the human spiritual world. The pandemic erosion to the human spirituality has increased the mental health crisis globally, and this erosion has seriously affected human behavior and increased suicidal ideation among individuals, especially among vulnerable groups: young people and women.

#### 3.1.1 Mental safety

Evidence suggests that personal safety concerns due to mental disorders will be the leading cause of global health-related burden by 2020, with depression and anxiety disorders being the primary manifestations (75). The emergence of the pandemic has created an environment that exacerbates many of the determinants of poor mental health. In a study examining the impact of the pandemic on the prevalence and burden of major depression and anxiety disorders worldwide in 2020, researchers found that the greatest increase in the prevalence of major depression and anxiety disorders in 2020 would occur in the regions most affected by the pandemic. The study estimated a 27.6% increase in cases of major depression, and a 25.6% increase in cases of anxiety disorders worldwide as a result of the pandemic. Of these, women were more likely to be affected by the pandemic than men, and younger people were more likely to be affected than older people (76). These trends have similarities in the Americas, Europe and Asia. For example, In Canada, researchers assessed depression and anxiety in 1,412 non-clinical adults from October 2018 to April 2022 (three pre-epidemic and seven post-epidemic) and found that depression and anxiety showed significant changes during the epidemic, with significant increases in depression and anxiety early in the epidemic (3). An assessment of levels and longitudinal changes in fear, anxiety, and depressive symptoms among 6,551 adults in Germany during the epidemic found that fear, anxiety, and depressive symptoms were significantly higher during the epidemic and that people with pre-existing anxiety, depression, and other psychiatric disorders were more vulnerable to exacerbated effects (18). A large online survey (*n* = 1,000) was conducted from 4 to 11 June in South Korea 2020. A series of regression analyses showed that depression was negatively associated with support for ‘everyday distance policies and stronger social distance policies (41). The prevalence of depression and anxiety after the pandemic is significantly higher than the 2.6 and 2.8% rates of depression reported in the Korean Community Health Survey in 2020 and 2018, respectively, and the 5.7% rate of anxiety reported in the Korean Mental Illness Survey in 2016 (42).

#### 3.1.2 Life safety

The impact of the COVID-19 pandemic on psychological and mental health could be far-reaching, and there also has some evidences that it is likely to lead to an increase in suicide rates, although this is not inevitable. As the epidemic spreads, suicide may become a more pressing issue. The rate of suicidal ideation during the pandemic is higher than that reported in studies of the general population before the pandemic, and may lead to higher suicide rates in the future (77). Suicide rates among survivors may increase during and after the pandemic, including mental health crises, which may last longer and peak later than the actual pandemic (78). In Bangladesh and Nepal, prevalence of suicidal ideation increases with the pandemic, with the effects lasting longer for women (43, 44). The economic environment of a country can play a determining role in medical assistance and social welfare, the better the social environment, the lower of exposure to life-safety threats. However, this is not absolute, suicide trends similar to those in underdeveloped countries have been observed in some developed countries during pandemics. From 2010 to 2019, the suicide mortality rate in Hungary decreased steadily, but this trend changed after the epidemic, with a significant increase in the suicide mortality rate in the post-epidemic period compared to the pre-epidemic trend: an overall increase of 16.7% (19). Compared to the average number of deaths in 2017–2019, the number of deaths in Poland in 2021 increased by 26.86%, with a higher increase in attempted suicides (20). In Croatia, the number of suicides increased significantly at the beginning of the epidemic in February 2020, with significantly more suicides recorded among married and unemployed people compared to the same period in 2019 (21). The Austrian government issued a blockade order from 16 March to 15 May 2020, and its strict measures may have had a serious impact on people’s mental health, leading to an increase in suicide attempts during the blockade period (22). In Japan, the suicide rate is highest in October 2020 (45). In the first 5 months of the epidemic (February to June 2020), the monthly suicide rate fell by 14%, which may be due to a number of complex reasons, including generous government subsidies, reduced working hours and school closures. In contrast, during the second wave (July to October 2020), the monthly suicide rate increased by 16%, with larger increases among women (37%) and adolescents (49%) (46).

Studies have shown that unemployment is one of the main reasons for the increase in suicide rates during the epidemic. In an experiment, the researchers predicted that a loss of 24.7 million jobs in the high scenario and 5.3 million jobs in the low scenario. In the high scenario, the global unemployment rate would increase from 4.936% to 5.644%, which would be associated with an increase in suicides of about 9,570 per year. In the low scenario, unemployment would rise to 5.088%, associated with an increase of about 2,135 suicides (79). In Sweden, researchers conducted a health survey of 1,558 people between February 2021 and February 2022 and found that unemployment and layoffs during the pandemic led to varying degrees of depression and anxiety, which are important mental safety factors that contribute to individual suicides (23). Factors that undoubtedly contributed to the increase in suicides during the epidemic also include a wide range of possibilities, and according to the study, in Lithuania and Poland (2,459 participants, 57.2% of respondents were female), the main factors for female suicides during the pandemic included a high level of environmental adaptation and loneliness, these factors are significantly correlated with suicidal tendencies (24).

#### 3.1.3 Teenagers show more vulnerability

The pandemic has had a significant impact on the mental health of teenagers, who are likely to be the group that suffers more during the epidemic. Suicidal behavior among adolescents increased by 35% during the pandemic, and suicide rates rose after an initial stabilization (80). Overall, the pandemic had a limited short-term effect on suicidal intention, but young people were more likely to report more severe suicidal ideation during the epidemic (81). Spanish researchers find significant increase in hospital admissions for suicidal behavior in adolescents during epidemic (25). Suicide is the leading cause of death among adolescents in the United States, and a proportion of young people have been significantly affected by the pandemic, leading to evidence of an increased risk of adolescent suicide (4). In Canada, students most at risk of suicide are those who are emotionally overwhelmed by the pandemic and unable to seek help (5). An online survey (including 509 Israeli citizens) shows that pandemic-related heart health complications are more common among the older adult, but that COVID-19 is also having a profound impact on the younger population (47). Using data from 1,109,776 Korean adolescents aged 13 to 18 years from the Korean Youth Risk Behavior Network Survey from 2005 to 2021 (for a nationwide series of cross-sectional surveys), found that during the pandemic period: sadness increased from 25.0% in 2020 to 26.6% in 2021, and the suicide rate: increased from 10.7% to 12.5% in 2021 (48). The study found that pandemics exacerbate the risk of suicide among adolescents through an online survey of 536 male and female students (age 21.46 ± 2.95 years) at Russian universities (49).

#### 3.1.4 Gender difference

During the pandemic, women suicide rates were higher than men, and were associated with depression, schizophrenia, alcoholism and other mental disorders. In Israel, the pandemic caused high levels of psychological distress in the general population. Observations of 587 participants showed that the mental health status of men and women in the general population in Israel was very different during the first blockade, with men and women likely to have different vulnerabilities to disaster-related stressors (such as those experienced during the pandemic), with younger women reporting more mental health problems (50). In assessing the range of psychopathological symptoms (anxiety, stress, depression, burnout) and their risk factors among frontline healthcare workers during the pandemic in the Russian Federation, the researchers found that the main factors contributing to the risk of psychopathology included: female and youth (51). A study of depression and anxiety symptoms during the pandemic in Australia found that younger age and being female were significantly associated with higher depression and anxiety scores (72). In France, there has been a significant increase in female suicide attempts since January 2021. Adolescent girls seem to be the most affected (26). In Korea, the total number of suicides during the pandemic did not differ from projections based on the pre-pandemic period. However, the number of suicides among women and women under 34 years of age was much higher than expected. The pandemic significantly increased suicides among women and young people (52). In Japan, compared to the average suicide rate from 2018 to 2019, the increase in women’s suicide rates increased more than men’s, with women experiencing the largest increase in October 2020, and people under 30 doing worse during the epidemic (53). It is worth noting that while women are more at risk of suicide overall, in some countries men may be more vulnerable. The suicide rates among Japanese men in 2020 increased in October and November compared to previous years (54). Suicide rates increased in India in the first year of the pandemic, with male suicides increasing most in 2020 compared to previous years (55). However, in Denmark, contrary to the rise in suicides is the proportion of self-inflicted injuries decreased for both women and men after the pandemic, but the decrease for men was smaller than that for women: 5.7% for women and 3.2% for men (27).

### 3.2 Family safety

While pandemic are a major global health threat, another global public health emergency—Domestic Violence (DV)—is becoming a growing challenge. Since the implementation of pandemic-related lockdown and physical distance, there has been a significant increase in cases of domestic violence globally. The pandemic poses a threat to the mental and life safety of individuals, who in turn transfer this threat to human safety, of which family safety is one. Policies such as curfews, lockdowns and restrictions during pandemics have resulted in people staying at home for longer periods of time than in the past, which, coupled with pandemic-induced job loss, perceived stress, and alcohol abuse, ultimately lead to the amplification of unstable relationships among family members and an increase in family safety issues.

#### 3.2.1 Domestic violence

An editorial published in BMJ in May 2020 discusses how the pandemic has amplified the rising incidence of domestic violence globally in two ways, and how the failure of health systems to respond adequately to domestic violence and abuse is a violation of human rights, compromising the health and well-being of survivors and their families. And it notes that while both men and women are affected, the incidence and severity is much higher in women (82). In Australia, a team of researchers from the Justice Centre at Queensland University of Technology (QUT) conducted a national survey which confirmed that the embargo is increasing the prevalence of domestic violence in Australia and around the world, based on survey data from 362 participants from the DFV sector (73). A survey of 602 married or cohabiting adult citizens of Trinidad and Tobago found an increase in perpetration of domestic violence (13%) and an increase in victimization of domestic violence (16%). The findings also indicated that males (17%) and females (13%) in the sample were more likely to be perpetrators of domestic violence, while males (25%) and females (12%) were more likely to be victims of domestic violence (6).

#### 3.2.2 Intimate partner violence

Domestic violence is a global public health problem that negatively affects physical and mental health, and intimate partner violence (IPV) is one of the most common forms. The pandemic contributed to an increase in the incidence and severity of IPV worldwide. Public health restrictions during the pandemic led to an increase in time spent at home with partners and a related increase in incidents of IPV, as evidenced by an increase in calls to hotlines and contact with other support services (83). European member states of the World Health Organization reported a 60% increase in emergency calls from women whose intimate partners had been violent during the pandemic. There was also a fivefold increase in the number of people accessing online violence prevention support lines, compared to the same period last year (84). A 23.38% increase in the incidence of IPV during the 3-month period of the Spanish blockade as a result of the pandemic (28). Palestinian men tend to show their vulnerability during pandemic outbreaks, leading to serious changes in the situation of domestic violence (56). India, significant increase in cases of domestic violence during the pandemic compared to previous years, higher morbidity in the early stages of the epidemic, pandemic affects women more severely than before (57). The number of victims of intimate partner violence in the US is on the rise from December 2019 to March 2022 (7). The types of intimate partner violence (IPV) victimization in the early stages of the US pandemic were primarily physical and sexual violence (8). During the pandemic, the level of violence against women in Spain has increased. In situations of incarceration, it is necessary to develop measures to protect women who lack social support and who live with the perpetrators of violence (29). During the UK COVID-19 blockade, the number of people seeking help for domestic violence (DV) and homicide increased. In addition, the blockade reduced opportunities for DV detection and disclosure through the suspension and remote provision of clinical services, continuing healthcare and other support services (30).

#### 3.2.3 Why family safety is exacerbated?

The strongest risk marker for IPV perpetration was loneliness, followed by anxiety symptoms, perceived stress, fear, boredom, substance use and lifestyle changes (85). Studies in Belgium have shown that victims of violence are more likely to be dissatisfied with their social interactions, and their weak social support and low trust in health services may lead to more incidents of violence during the pandemic (31). In the United States, a social survey of 13,597 female participants found that IPV experience was associated with poorer sleep quality, shorter sleep duration, and increased alcohol consumption, and that IPV experience at the onset of the pandemic was associated with more severe mental health symptoms and modifiable health factors in female participants under the age of 60 (9). IPV is significantly associated with unemployment, women who do not work and whose partners do not work have higher levels of emotional violence during the pandemic in Turkey (32, 33). In India, unemployment motivates domestic violence perpetrators during restrictions (58). And there are studies showing that the end of the embargo does not necessarily mean a rapid decline in IPV. The incidence of IPV may have increased as a result of the economic fallout. This is particularly worrying as economic stress increases most types of IPV. Particular attention should be paid to couples who have not been exposed to violence before, who have children and who are of lower socio-economic status, as these couples are most affected (86).

#### 3.2.4 Women and children suffer more

During the pandemic, stay-at-home policies were implemented worldwide. However, there is growing concern that such policies may increase violence against women. Domestic violence, including intimate partner violence, is a pandemic that occurred in conjunction with COVID-19. During the pandemic, a large percentage of women who experienced partner violence had to live with their abusers. The incidence of domestic violence is rising rapidly, particularly affecting women and children.

In Nigeria, the conditions of the pandemic blockade not only create opportunities for motivated perpetrators, but also increase women’s vulnerability to sexual victimization (63). And the prevalence of IPV was 57.5% in women compared to 42.5% in men, and IPV was significantly associated with younger age (64). In Colombia, recent statistics on domestic violence highlight the need to reorganize the national public health system and adopt effective strategies and emergency plans to mitigate the impact of COVID-19 on the mental health of women and children throughout the country (10). In Peru, where a strict nationwide blockade has been in place since mid-March, nearly 60% of women had experienced violence prior to COVID-19. Using telephone administrative data, researchers found that the incidence of calls to a domestic violence helpline increased by 48% between April and July 2020, with the impact increasing over time (11). A Belgian study on domestic violence found a significant increase in the incidence of psychological aggression among women during the epidemic (34). In Tunisia, violence against women increased significantly during the blockade (from 4.4% to 14.8%), with psychological abuse being the most common form of violence (96%). The vast majority of those who experienced violence during the blockade did not seek help, and women who were abused before the blockade were at increased risk of violence (65). In Turkey, the pandemic process increases women’s vulnerability to intimate partner violence (IPV), and as the frequency of IPV increases, women’s perceptions of stress also increase (35). In Iraq, there has been a significant increase in the humiliation, forced sexual intercourse and intimidation of women during the blockade (59). Violence against women in Jordan during COVID-19 was as high as 40%, with unemployment as the main predictor (60). According to data, the risk of and vulnerability to violence against women and girls in Uganda has increased significantly since the outbreak of the pandemic (66). Pandemic mitigation measures such as curfews, lockdowns and movement restrictions can be effective in reducing the spread of epidemics. However, they can also lead to sexual violence. Using data from the Kenya Health Information System and various time-series methods to model the unintended consequences of pandemic mitigation measures on trends in sexual violence in Kenya, researchers found that model-dependent increases in reported sexual violence ranged from 73% to 122%, mainly among 10 to 17-year-olds (67).

Fortunately, some countries have begun to pay attention to this problem and have taken effective measures. For example, in Japan, four phone lines across the country will be open 24 h a day, and the Cabinet Office will continue to allocate $3.6 million for domestic violence counseling during the pandemic (87). As pandemic preparedness activities increased the incidence of intimate partner violence (IPV) and negatively affected access to health and legal systems, this indirectly contributed to the establishment of IPV services in Kenya and Malawi, enabling a significant proportion of the population to access IPV services during the pandemic (68). During the pandemic, the New Zealand government provided housing for women experiencing domestic violence in New South Wales to help them escape domestic violence (74).

### 3.3 Social safety

Pandemics pose a threat to the mental and life safety of individuals, who in turn transfer this threat to human safety, with family safety being one scenario and social safety another. In this review, the impact of pandemic on social safety mainly includes assaults, thefts, burglaries, robberies, car thefts, homicides, gun violence, drug trafficking, racism, cybercrime, piracy incidents and so on. In social safety, pandemic-induced victimization extends from individuals and families to social members, with a wider range of impacts and diverse manifestations.

#### 3.3.1 Conflicting crime trends

Governments adopts strict social bans during the pandemic, which hindered the spread of the virus while also restricting people’s freedom of movement, and indirectly, had an impact on human crimes. Common sense would suggest that the pandemic’s contribution to the decline in crimes is inevitable (the macro data also proves it), for example, social bans impose strict street control and require people to stay at home, which prevents criminal behavior and thus reduces social crimes. However, as mentioned above, human safety is multiple, overlapping and interconnected, the review found that in terms of micro-evidence, the trend of pandemics leading to a decrease in crime rates did not apply in all cases, but rather that specific types of crime increased during pandemics.

#### 3.3.2 Decreased crime

Fear of the dangers posed by the virus has made people more cautious about going out, and this, combined with a home policy that reduces the environmental opportunities for crime, has contributed to a downward trend in the general crime rate and a significant increase in people’s sense of social safety. Using police data on daily crime counts, Nivette and colleagues (88) examined the impact of stay-at-home restrictions on assaults, thefts, burglaries, robberies, car thefts and homicides in 27 cities in 23 countries in the Americas, Europe, the Middle East and Asia. The main conclusion was that global crime rates fell by 37% after governments introduced curfew restrictions. An estimate of the impact of pandemic outbreaks on crime, based on data from 25 major US cities, found that Pittsburgh, New York, San Francisco, Philadelphia, Washington, D.C. and Chicago saw crime rates fall by at least 35% (12). In Japan, the 2020 pandemic resulted in a 12.7 and 20.9% reduction in the victimization rates per 100,000 population for violent and property crime, respectively. The researchers also found that intentional crimes, such as burglary and sexual assault, declined more than non-intentional crimes, such as homicide (61). A study examining the impact of curfews on female homicides in Turkey found that the probability of a woman being killed by an intimate partner fell by around 57% during strict social distance, and by 83.8% during curfews, compared to the same period in 2014–2019 (36).

#### 3.3.3 Increased crime

Stay-at-home restrictions are associated with significant reductions in crime rates, but there is wide variation across cities and types of crime. Gun violence in the United States increased by 30% from pre-pandemic levels in 2020–2021 (13). The recent surge in gun sales during the pandemic has been accompanied by a sustained increase in shootings, injuries, fatalities in the period after the pandemic reopened compared to historical years. Gun violence, injuries and deaths increased after the pandemic reopened. In addition, mass shootings increased despite the relative calm initially brought about by the pandemic. This suggests that the “re-opening” exacerbated an already serious national gun epidemic (89). Although crimes related to domestic violence, burglary and car theft fell dramatically in Mexico during the pandemic, crimes related to robbery, kidnapping and homicide were unaffected and organized crime remained stable (14). In Brazil, extortion, theft and robbery fell by at least 41.6% after the pandemic. However, the researchers found no change in organized crime (15). Where there is evidence that nighttime crime rises, and strict measures in the wake of the pandemic may also have led to an increase in piracy incidents in Nigeria (69, 90). In Norway, restrictions and bans on alcohol consumption reduced crime during the pandemic, but when bars were told not to sell alcohol after midnight, there appeared to be an unexpected increase in crime (37). The frequency of total arrests for illicit drug trafficking in Bangladesh showed a sharp upward trend and was 75% higher than expected during the pandemic (62). Sweden, significant falls in total, assault, pickpocketing and burglary, but no change in drug offenses (91). In Canada, all types of crime changed significantly during the pandemic compared to metropolitan areas, with increased crime rates varying across neighborhoods (92). In England and Wales, researchers found that crime in most small areas remained stable throughout the epidemic (38).

The young generation appears to have been involved in more crime during the pandemic. Evidence suggests that pandemic leads to a significant increase in the number of shootings involving young people in the United States 2020 (16). Shootings vary by population. Preschool exposure was lowest for white children and highest for black children (who were 4.44 times more exposed to neighborhood gun violence than white children). The pandemic increased exposure by 27% among the lowest risk population (i.e., white children), but the impact of the pandemic was greater for almost all non-white children. Baseline levels of violence and racial disparities varied widely by region, with the highest levels in the South. There were large racial disparities in children’s exposure to neighborhood gun violence, and these disparities widened during the pandemic (93). Zimbabwe has experienced a dramatic increase in the use of illicit drugs during the pandemic, and of particular concern is the upward trend in the use of drug substances among young people (94). Evidence points to a significant increase in the rate of juvenile property-related crime in Israel during the blockade period, compared to other periods (70).

Racism has always plagued human solidarity, and the pandemic has worsened this division. In the United States, the conditions created by the pandemic have exacerbated existing inequalities in communities of color, putting them at risk of violence. Racism against people of Asian descent increased by more than 300% after the pandemic, with one in five Asian Americans reporting a direct experience of overt discrimination, and it has been an important factor in the psychological distress and suicidal tendencies of Asian and Asian American students (17, 95). A large proportion of the Ugandan population were victims of discrimination during the pandemic, most at the hands of law enforcement officials (71).

Stay-at-home policy have prompted people to rely on the Internet more than ever before, which indirectly facilitates cybercrimes. Disruptions to people’s real-life patterns can affect cybercrime levels, with blocking measures helping to reduce opportunities for predatory crime in physical spaces, while at the same time people are spending more time connected to the internet, which can lead to increased opportunities for cybercrime. For example, in the UK, growth in both total cybercrime and total fraud has exceeded predicted levels. Particularly online shopping and auction fraud, and social media and email hacking (the two most common types of UK cybercrime) (39, 40).

## 4 Future trends

With the development of human society, human safety is becoming increasingly interconnected, and the impact of pandemic deepens the multidimensional uncertainty of human safety. As an obstacle to social development, the pandemic not only poses an immediate threat to individual safety, but also deals a severe blow to the safety of families and society. The pandemic has deepened the fissures of existing human safety and opened up new abysses. In order to address these threats, humanity needs to be more united. In particular, it is necessary to strengthen the extension research in the following aspects.

### 4.1 Human needs

When the concept of human safety is combined with Maslow’s hierarchy of needs, it may be possible to better explain the changes in human safety that occurred during the pandemic. In Maslow’s hierarchy of needs, human needs are divided into physiological (basic survival needs), safety (social and occupational safety), social, esteem and self-actualization needs (96). Maslow’s model suggests that in order to achieve higher-level goals (e.g., love and belonging, respect, and self-actualization needs), individuals must first address life-safety needs and then higher-level needs. This review believes that the pandemic embargo policy is undermining human safety needs, such as personal safety, which is suffering both mentally and physically, the disruption of family cohesion, and the increase in certain types of societal crime, which are included in Maslow’s hierarchy of human needs, such as safety, love, respect, and self-actualization, by incorporating Maslow’s model, it can help reveal the deeper phenomena in global human safety crises such as COVID-19. It’s important to understand that Maslow’s model is not linear and monolithic, policy makers need to consider not only the impact of the pandemic on human physiological needs (human survival during a public health event), but also incorporate safety, love, respect and self-actualization into the policy model to address the COVID-19-related crisis, or else unnecessary and long-term social harm and instability will result.

### 4.2 Women’s insafety

Gender differences show that women suffered more during pandemic. The pandemic increased women’s vulnerability. The human needs, such as safety, respect, love and self-fulfillment were reduced during the pandemic. It is worth noting that apart from the fact that there are few gender differences in the impact of pandemic on human survival (virus infection), women are likely to have suffered more after the pandemic globally. First, the pandemic exacerbated women’s exposure to family and society injustice, however, women were already more vulnerable before the pandemic. Second, men in most countries showed greater vulnerability during the pandemic (that is strangely similar in developed, developing and underdeveloped countries), and men are transferred this vulnerability to women, deepened the scars in the household. Finally, if women do not receive targeted care from society, their vulnerability may be passed on to the next generation, as they have greater social, legal and moral responsibility as the main actors in the education of the next generation.

### 4.3 Human resilience

The direct impact of pandemics on human safety has been dramatically reduced as global health cooperation, and public health researchers have made significant advances in this area, providing a foundation for future human safety. However, the impact of a pandemic on people’s mental, family and social health is likely to be a far-reaching and long-lasting process that will require interdisciplinary research and deeper pioneering by researchers to build human resilience. In particular, by using interdisciplinary approaches to understand the complex mechanisms underlying human resilience, by using advanced technologies to develop scalable and effective interventions. For example, developing resilience interventions for target populations, environments and socio-cultural contexts. To achieve this, we need to take an interdisciplinary view of human resilience development and take initiatives to increase resilience recovery across multiple dimensions of human safety. Concerted efforts are needed not only in the short term to respond to emerging challenges (e.g., COVID-19 pandemic), but also in the long term to strengthen pandemic preparedness, improve mental health and promote social cohesion (96).

## 5 Discussion

This review found the increase in suicide during pandemic is a global trend. Whether in developed countries such as Hungary or in developing countries such as Bangladesh, there is a clear upward trend in suicide rates (19, 43). While better social policies in developed countries may slow the rise in suicide rates in the short term, but in the long term, the upward trend is irreversible. The evidence from Japan and Nepal (44, 46) shows that the significant increase in suicide rates following the pandemic occurred at roughly the same time in both developed and underdeveloped countries (July, August, September 2020). In terms of gender differences, there is evidence of an upward trend in male suicide rates in India in 2020 (55), and a downward trend in male self-inflicted injuries in Denmark (27). While overall global crime declined during the pandemic (12), there is additional evidence that this finding is not universal. For example, there was a significant increase in gun crime in the United States (13), signs of an increase in piracy in Nigeria (90), and a sharp upward trend in the overall frequency of arrests for illicit drug trafficking in Bangladesh (62). It is also important to note that inequality and discrimination are pervasive social problems around the world. A study in Uganda found that a large proportion of the Ugandan population experienced discrimination during the pandemic (71), which may be due to the low level of education *per capita* in underdeveloped countries. But the truth is that the pandemic is also exacerbating inequalities between people of color in developed countries such as the United States (17, 95).

Human safety was hard hit during the epidemic. This impact is not only seen in the safety of human life (life-threatening), but also in the greater negative impact on human psychology. During the pandemic, individuals are not only exposed to viral aggression in their biological systems (which in most cases is innate), but also face significant challenges to their mental endurance and psychological resilience (which in most cases is nurtured). During the pandemic, household safety vulnerabilities may be an indirect consequence of personal safety challenges. According to the World Health Organization that household safety risk was destabilized before the pandemic, and as the pandemic spread, the fissures in safety caused by this risk widened (75). The concept of societal safety is shaped by the combination of human actions and linkages, and when individual actions and linkages are threatened, the societal safety architecture is destabilized (2). Furthermore, as the operational structure of a society changes, new safety threats may emerge.

Some interesting contrasts were found in the study, while journal such as Nature Human Behaviour (NHB) has published findings on the decline in global crimes during COVID-19 (88), there are also findings in journal such as The BMJ that suggest that pandemics instead promote certain types of crime (13). In addition, both authoritative and individual studies have highlighted the contribution of pandemics to human depression and anxiety, and that impacts on the mental level can lead to life-threatening behaviors. Most studies have highlighted that more victims of pandemic spin-offs are likely to be adolescents and women, and have analyzed the causes of such effects, but there has been a lack of in-depth research on mitigation and prevention, which could be a focus for future research in this area.

## 6 Limitations

This narrative review has several limitations. First, generalized descriptions of the impact of the pandemic on the areas of personal safety, family safety, and social safety were limited to the literature cited in the articles (only 40 countries were included). Second, although the review included mainstream literature in relevant areas after the pandemic, the cited literature was not screened according to the quality of the studies and reporting mechanisms, and the screening process incorporated the subjective judgment of the author, which can be biased, that is also unavoidable in review studies (97). Then, after the pandemic, the author did not compare this review with similar reviews to highlight the strengths and weaknesses due to the lack of relevant literature in this area. In addition, when combined with Maslow’s hierarchy of needs, the focus of this review is on higher human safety needs other than survival, so the literature related to the survival safety is not included in the topic of new challenges to human security.

## 7 Conclusion

Based on this review, it may be concluded that COVID-19 exacerbated human safety threats and rift, individual insafety ultimately leads to more serious social insafety. While societal crime rates show a downward trend during the pandemic, however, specific types of crime, such as drugs, firearms and organized crimes, increased. Pandemics provide a fertile breeding ground for domestic violence, which indirectly contributes to the attention given to the family safety. Women and children show more vulnerability to global disasters. In addition, COVID-19 exacerbates discrimination and suicidal behaviors globally. The aim of this review is to understand the changes in human safety during the pandemic through a global review, to increase the comprehensibility of the concept of human safety, to draw attention to the invisible impacts of the pandemic, and to increase the focus of future research on higher-level safety needs beyond the need for survival.

## Author contributions

SA: Writing – original draft, Writing – review & editing.
